# Relationship of life expectancy with quality of life and health-related hope among Japanese patients receiving home medical care: The Zaitaku Evaluative Initiatives and Outcome Study

**DOI:** 10.1371/journal.pone.0295672

**Published:** 2023-12-14

**Authors:** Masakazu Yasunaka, Yukio Tsugihashi, Shinu Hayashi, Hidekazu Iida, Misaki Hirose, Yutaka Shirahige, Noriaki Kurita, and the ZEVIOUS group

**Affiliations:** 1 Dr. Net Nagasaki, Nagasaki-City, Nagasaki, Japan; 2 Yasunaka Neurosurgery Clinic, Nagasaki-City, Nagasaki, Japan; 3 Medical Home Care Center, Tenri Hospital Shirakawa Branch, Tenri-City, Nara, Japan; 4 Department of Public Health, Health Management and Policy, Nara Medical University, Kashihara-City, Nara, Japan; 5 You Home Clinic, Bunkyo-ku, Tokyo, Japan; 6 You Home Clinic Heiwadai, Nerima-ku, Tokyo, Japan; 7 Department of Clinical Epidemiology, Graduate School of Medicine, Fukushima Medical University, Fukushima-City, Fukushima, Japan; 8 Center for Next Generation of Community Health, Chiba University Hospital, Chiba-City, Chiba, Japan; 9 Hirose Clinic, Nagasaki-City, Nagasaki, Japan; 10 Shirahige Clinic, Nagasaki-City, Nagasaki, Japan; 11 Department of Innovative Research and Education for Clinicians and Trainees (DiRECT), Fukushima Medical University Hospital, Fukushima-City, Fukushima, Japan; 12 Center for Innovative Research for Communities and Clinical Excellence (CiRC^2^LE), Fukushima Medical University, Fukushima-City, Fukushima, Japan; Transilvania University of Brasov: Universitatea Transilvania din Brasov, ROMANIA

## Abstract

Spiritual care for patients’ quality of life (QOL) and hope should be included in home medical care for patients with limited life expectancy. This study aimed to analyze the associations between estimated life expectancy, QOL, and hope among patients receiving home medical care in Japan. This multicenter cross-sectional study involved 29 home medical care facilities in Japan. Patients were categorized by estimated life expectancy, as assessed by home medical care physicians. The outcomes were QOL measured via the Quality-of-Life Scale for Elderly Patients Receiving Professional Home Care (QOL-HC: higher score indicates better QOL), the domain scores of health-related hope (“*health*,” “*role and connectedness*,” and “*something to live for*”; higher scores indicate higher levels of hope), and life functioning measured using the WHO Disability Assessment Schedule 2.0 (WHODAS 2.0; higher score indicates worse functioning and disability). QOL-HC scores were significantly higher in patients with shorter life expectancy (< 6 m vs. ≥ 1 y, adjusted mean differences: 0.7 points [95%CI 0.1 to 1.3]). Regarding health-related hope, “something to live for” scores were associated with shorter life expectancy (< 6 m vs. ≥ 1 y, -17.7 points [-34.2 to -1.2]), whereas “role and connectedness” scores did not change remarkably with shorter life expectancy (< 6 m vs. ≥ 1 y, -3.3 points [-16.4 to 9.8]). Furthermore, shorter life expectancy was associated with higher WHODAS 2.0 scores (< 6 m vs. ≥ 1 y, 19.6 points [4.3 to 34.8]). Home medical care physicians who engage in spiritual care should facilitate thoughtful dialogue with their patients by recognizing declines in life functions and hope for fulfilment, which are associated with short life expectancy.

## Introduction

Patients in Japan who require home medical care have impaired physical functions that render them unable to visit physicians [1, 2]. The leading causes vary, and include difficult-to-cure conditions such as cancer, multiple comorbidities including dementia and cerebrovascular disease, and neuromuscular diseases with severe disabilities [2–4]. Therefore, when providing care to these patients, their lives at home should be supported in accordance with their preferences and needs [1, 5, 6]. Other priorities include the maintenance of quality of life (QOL), covering social and role functioning [7, 8], and relieving psychological distress [6]. For patients with progressive illnesses, hope is a psychological state considered an inner resource and essential coping strategy for maintaining QOL [9]. Coping and fostering hope are also considered central to clinical practice in palliative care [10]. However, it remains unclear how hope and QOL evolve for patients receiving home medical care who typically desire to stay home until the end of their lives.

Hope involves the discovery of meaning in life (such as relationships and a sense of connection with life and others) [11] and the pursuit of future goals; moreover, along with QOL, it is closely associated with the well-being of patients with limited life expectancy [12]. For example, the following question from a patient with advanced cancer—“Doctor, is there a chance of cure?”—is connected to hope regarding a cure [13]. Furthermore, medical care providers are concerned that explicitly informing the patient of the lack of curative treatment and inevitable death may cause despair [5, 14]. Although multiple studies have reported an association between the progression of cancer and loss of hope [15], the association between remaining life expectancy and hope has not been investigated in home medical settings. One reason for the paucity of psychometric studies of hope among individuals with a limited life expectancy is that a generally accepted construct of hope in psychology—the belief that one can find a way to achieve one’s wishes and the belief that one will keep moving toward the achievement of one’s wishes [16] - is difficult to apply to those who have a limited ability to function independently. Therefore, the health-related hope scale, which was developed based on the construct of belief to find and achieve wishes related to "health & illness," " role & social connectedness," and "something to live for" for patients with chronic illnesses [17], could be applied to the aforementioned individuals.

Providing goal-oriented care aligned with patients’ preferences and needs is expected to improve QOL among older patients with terminal cancer and advanced heart failure [9, 18]. It has also been emphasized as the benchmark for long-term care, including home medical care provided for older patients [7, 8, 19]. However, few studies have examined the association between limited life expectancy, QOL, and hope in diverse populations receiving home medical care. Clarifying these associations enables home medical care physicians, who are in a unique position to directly provide palliative care by visiting patients where they live [20, 21], to discuss the goals and values of care with their patients to maintain hope and QOL.

Therefore, this study aimed to analyze the associations between estimated life expectancy, QOL, and hope using data from a multicenter cross-sectional study conducted among patients receiving home medical care in Japan.

## Methods

This study was conducted as part of the Zaitaku Evaluative Initiatives and Outcome Study (ZEVIOUS), which is a multicenter cross-sectional survey conducted in Japan between January and July 2020. Twenty-nine home medical care facilities in the Tokyo Metropolitan area, Nara, and Nagasaki Prefectures were included in the study. In Japan, home medical care is usually provided by primary care physicians or hospitalists who are primarily engaged in such services [22], so we will use the term "home medical care facilities" for facilities that have the functions to provide home medical care services. Subspecialty certification is not required to provide home medical care: palliative care training prior to home medical care is left to individual physicians, and for example, the use of sedatives for end-of-life care in home medical settings is determined by the physician’s experience. Patients were eligible for the study based on the following criteria: (1) those who were receiving continuous home medical care from home care physicians at the participating facilities, regardless of the length of their care, and (2) those who were judged by their physicians to be capable of responding to the questionnaire survey [22]. Exclusion criteria were patients who were considered by their physicians to be unable to complete the questionnaire due to cognitive function, mental status, or physical reasons [22]. The participants signed the written consent form and completed the questionnaire at their residence. Patients unable to write due to visual or physical impairments were allowed to complete the form with help from a family member or formal caregiver. The forms were mailed directly to the central research office to ensure that the completed questionnaires were not seen by the physicians treating the patients. This study was approved by the Institutional Review Board of Fukushima Medical University. Additionally, it was conducted in accordance with the principles of the Declaration of Helsinki and the Ethical Guidelines for Medical Research issued by the Japanese Ministry of Health, Labor, and Welfare [23, 24].

### Exposure

The home medical care physician assigned to the patient answered the following question: “How long do you expect the clinical prognosis (life expectancy) of this patient to be?” The physician was allowed to choose from five options: “less than one month,” “more than one month to less than three months,” “more than three months to less than six months,” “more than six months to less than 12 months,” and “more than 12 months.” Considering the frequency of the obtained responses, we merged them into the following three categories during the analysis phase: “less than six months,” “more than six months to less than 12 months,” and “more than 12 months.” These responses also considered a question ‘Would I be surprised if the person in front of me was to die in the next six months or one year?’, which was proposed in a prior study for initiating a discussion about end-of-life care needs and preferences in the United Kingdom [25].

### Outcomes

The following three constructs were measured as the outcomes in this study: the Quality-of-Life Scale for Elderly Patients Receiving Professional Home Care (QOL-HC), the Health-Related Hope Scale (HR-Hope), and the World Health Organization Disability Assessment Schedule 2.0 (WHODAS 2.0). This study used the validated Japanese versions of the scales.

1. The QOL-HC: This four-item questionnaire assesses the QOL of older patients receiving home medical care [7]. The face validity of the QOL-HC was ensured through the derivation of items by physicians and care managers and deliberate item selection by geriatricians (S1 Table). Each item is rated on a 3-point scale ranging from “*never agree”* (0 points) to “*always agree”* (2 points), with a total score ranging from 0 to 8 points. Higher scores indicate better quality of life in patients receiving home care.

2. The HR-Hope Scale: This 18-item uni-dimensional scale assesses hope related to health among persons with chronic conditions (S2 Table) [17]. Through structural validation, the following three subdomains are scored: “something to live for” (five items), “health and illness” (six items), and “role and connectedness” (seven items). Responses to each item are rated on a four-point Likert scale ranging from 1 = *I don’t feel that way at all* to 4 = *I strongly feel that way*. After obtaining the average score for the total domain and each subdomain, the scores were scaled from 0 to 100. Patients without family were exempted from answering two items in the “role and connectedness” subdomain. Higher overall and subdomain scores indicate better hope, which is perceived by the patients themselves.

3. The WHODAS 2.0: This 12-item scale measures functioning and disability, regardless of the disorders that cause dysfunction or disability, while complying with the International Classification of Functioning, Disability, and Health principles (S3 Table) [26]. It includes the following six domains: knowledge and communication (cognition), mobility, self-care, getting along with people (socializing), daily activities, and engagement in society. Additionally, this scale includes items on challenges experienced over the past 30 days. The Japanese version of the 12-item WHODAS 2.0 (S3 Table) has demonstrated excellent internal consistency reliability (coefficient alpha = 0.93) [27]. Each item is scored on a 5-point scale ranging from “*none”* (1 point) to *“extreme/cannot”* (5 points), with the total score for each item converted to a scale of 0 to 100. Higher scores indicate worse functioning and disability, which is subjectively recognized by the patients.

### Other variables

Age, sex, educational attainment, presence of family members, and comorbidities were also recorded. The attending physician was asked to provide the comorbidities resulting in home medical care. Multiple choices were allowed, and other variables were collected using a patient questionnaire.

### Statistical analysis

Statistical analyses were performed using Stata/SE version 15 (StataCorp, College Station, TX, USA). Patient characteristics were described by means and standard deviations for continuous variables, and by frequencies and percentages for categorical variables for the overall and expected prognoses. The QOL-HC, HR-Hope, and WHODAS 2.0 scores were similarly described for overall and expected prognoses. Mixed-effects linear regression models were performed with consideration of clustering effects by facility to evaluate the relationships between expected prognosis and the QOL-HC, HR-Hope, and WHODAS 2.0 scores. For the QOL-HC analysis, we used robust variance estimation because the scale did not meet the standard assumptions of equal variances and normality. Age, sex, educational attainment, family, and comorbidities were entered into the models as covariates. In addition to the general linear models, other models were constructed by treating the expected prognosis as a continuous instead of a categorical variable to test the trends of monotone relationships between the expected prognosis and outcome variables [28]. Missing covariates were imputed using a multiple-imputation approach with chained equations. Statistical significance was defined as *p* < 0.05.

## Results

**Fig 1:**
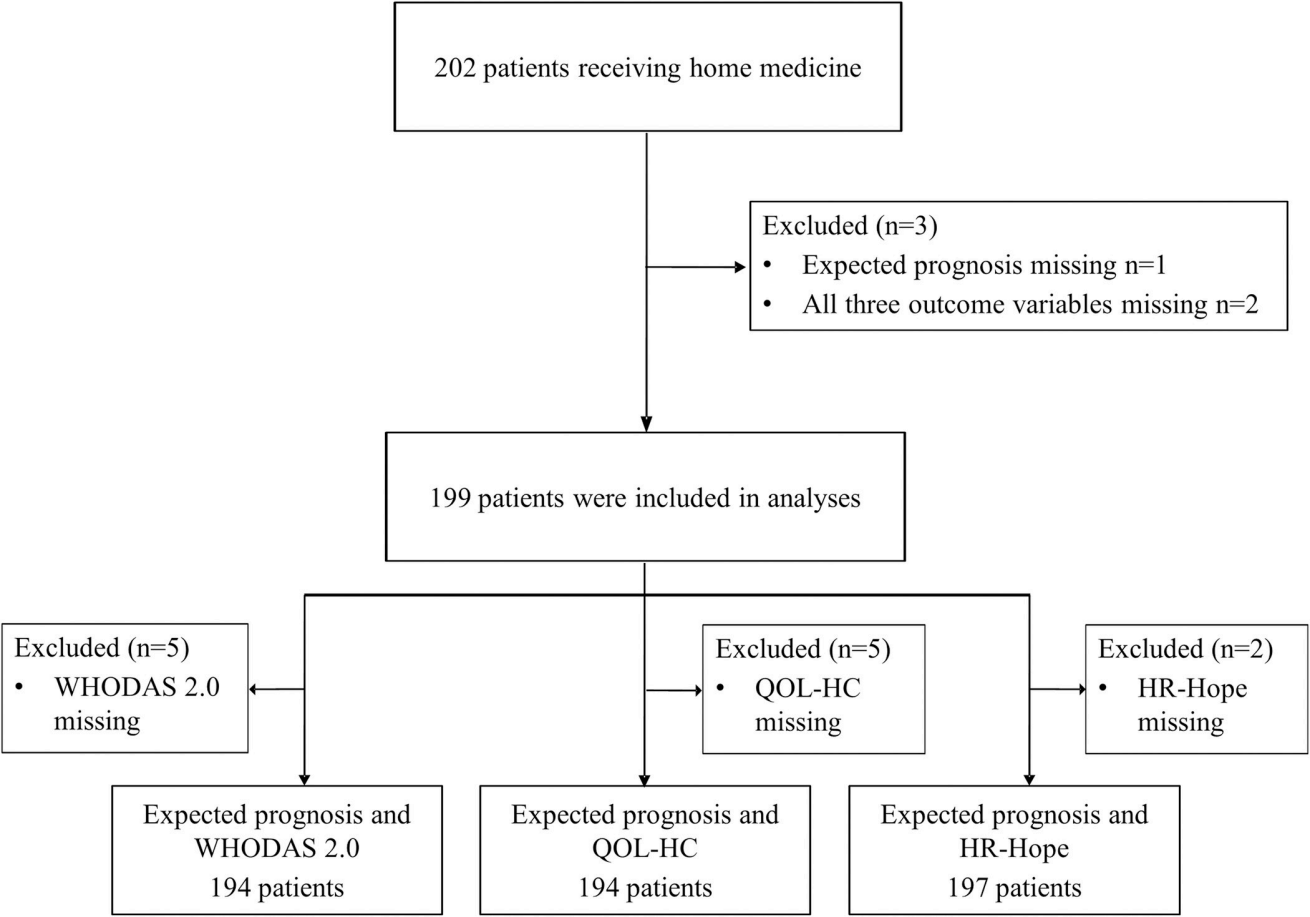
Flow diagram of the participants.

Of the 202 patients examined in this study, we excluded one patient without an expected prognosis and two patients who did not provide data for all three outcome variables. Consequently, data from 199 patients were used to investigate the associations between the expected prognosis and QOL-HC, HR-Hope scores, and WHODAS 2.0 (Fig 1) (S4–S6 Tables). Table 1 presents patient characteristics. The mean age (standard deviation) was 80.0 (14) years; 117 (58.8%) participants were women. The comorbidities for which home medical care was required varied. Notably, cerebrovascular disease (*n* = 30, 18.4%), articular disease (*n* = 26, 16%), dementia (*n* = 34, 20.9%), neuromuscular disease (*n* = 22, 13.5%), and spinal cord injury (*n* = 7, 4.3%) were the most common among patients with expected prognoses of ≥ 12 months, and malignancy was the most common (*n* = 7, 58%) among patients with expected prognoses of < six months.

**Table 1:** Characteristics of 199 patients separated by expected prognosis^a^ (2020).

	Expected prognosis			Total
	≥*12 mo*	*≥6-<12 mo*	*<6 mo*	
	n = 163	n = 24	n = 12	n = 199
**Demographics**				
**Age, yr**	79.8 (14.5)	81.5 (12.5)	80.5 (9.6)	80 (14)
**Women, n (%)**	92 (56.4%)	15 (62.5%)	10 (83.3%)	117 (58.8%)
**Education, n (%)**				
*Elementary school or junior high school*	57 (35.6%)	7 (30.4%)	4 (33.3%)	68 (34.9%)
*High school*	51 (31.9%)	8 (34.8%)	4 (33.3%)	63 (32.3%)
*College*, *university*, *or graduate school*	52 (32.5%)	8 (34.8%)	4 (33.3%)	64 (32.8%)
*missing*, *n*	3	1		4
**Presence of family, n (%)**	143 (87.7%)	20 (83.3%)	12 (100%)	175 (87.9%)
**Comorbidities, n (%)**				
Cerebrovascular disease	30 (18.4%)	3 (12.5%)	1 (8.3%)	34 (17.1%)
Heart disease	46 (28.2%)	11 (45.8%)	3 (25%)	60 (30.2%)
Malignancy	12 (7.4%)	8 (33.3%)	7 (58.3%)	27 (13.6%)
Respiratory disease	25 (15.3%)	6 (25%)	3 (25%)	34 (17.1%)
Articular disease	26 (16%)	1 (4.2%)	0 (0%)	27 (13.6%)
Dementia	34 (20.9%)	4 (16.7%)	0 (0%)	38 (19.1%)
Neuromuscular disease	22 (13.5%)	1 (4.2%)	0 (0%)	23 (11.6%)
Fracture/Fall	17 (10.4%)	3 (12.5%)	0 (0%)	20 (10.1%)
Weakness	27 (16.6%)	9 (37.5%)	1 (8.3%)	37 (18.6%)
Spinal cord injury	7 (4.3%)	0 (0%)	0 (0%)	7 (3.5%)

^a^Means (SD) are presented for continuous data

Weakness: weakness associated with advancing age

Table 2 shows the summary for the QOL-HC, HR-Hope, and WHODAS 2.0 scores for the overall patient population and expected prognosis subgroups. The means of the QOL-HC and WHODAS 2.0 scores among patients with an expected prognosis of < six months were higher than among those with an expected prognosis of ≥ 12 months. In contrast, the means of the HR-Hope overall and subdomain scores were lower among patients with an expected prognosis of < six months.

**Table 2:** Description of outcomes separated by expected prognosis^a^ (n = 199).

	Expected prognosis			Total
	≥*12 mo*	≥*6-<12 mo*	*<6 mo*	
	n = 163	n = 24	n = 12	n = 199
**QOL-HC, pts**	6.4 (1.5)	6.6 (1.2)	6.9 (1.3)	6.4 (1.4)
*missing*, *n*	5			5
**HR-Hope**				
**Total, pts**	57.6 (22.6)	59.7 (24.9)	48.3 (25.7)	57.3 (23.1)
**Something to live for domain, pts**	57.2 (26.2)	58.9 (29.6)	41.7 (27)	56.5 (26.8)
*missing*, *n*	1			1
**Health domain, pts**	53.7 (27)	59.5 (23.6)	43.1 (31.5)	53.7 (27)
*missing*, *n*	1	1		2
**Connectedness domain, pts**	60.7 (21)	62.8 (24)	57.5 (21.5)	60.8 (21.4)
**WHODAS 2.0, pts**	51.1 (25.4)	54.5 (29.2)	65.1 (31.2)	52.3 (26.4)
*missing*, *n*	4	1		5

^a^Means (SD) are presented for continuous data.

WHODAS 2.0: WHO Disability Assessment Schedule 2.0, HR-Hope: health-related hope, QOL-HC: QOL for patients receiving home-based medical care

**Fig 2:**
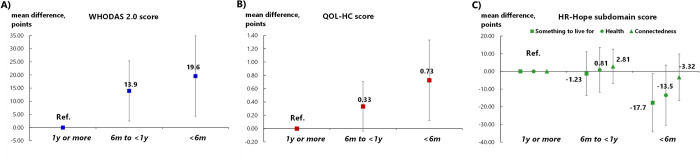
Associations between life expectancy and living function, quality of life, and health-related hope. A. Association between life expectancy and living function measured via the World Health Organization Disability Assessment Schedule 2.0: Blue squares represent point estimates. B. Association between life expectancy and quality of life for home medical care measured by the Quality-of-Life Scale for Elderly Patients Receiving Professional Home Care: Red squares represent point estimates. C. Associations between life expectancy and hope measured via the Health-Related Hope Scale: Green squares, circles, and triangles represent point estimates for something to live for, health, and role and connectedness, respectively. Estimates were derived from linear mixed models including age, sex, educational attainment, presence of family members, and comorbidities (cerebrovascular disease, heart disease, malignancy, respiratory disease, articular disease, dementia, neuromuscular disease, fractures or falls, weakness, spinal cord injury) as covariates.

Fig 2 and S4 Table present the association between the expected prognosis and QOL-HC scores. Compared to expected prognoses of ≥ 12 months, those of < six months were associated with higher QOL-HC scores (0.7 points [95%CI 0.1–1.3]); expected prognoses of six to 12 months were not statistically associated with greater QOL-HC scores (0.3 points [95%CI -0.04–0.7]). However, an increasing trend was observed in QOL-HC scores as the expected prognosis decreased (*p* for trend = 0.006). Fig 2 and S5 Table present the associations between the expected prognosis and HR-Hope subdomain scores. Compared to expected prognoses of ≥ 12 months, those of < six months were associated with lower “something to live for” scores (-17.7 points [95%CI -34.2 to -1.2]). However, evidence of a decreasing trend in the “something to live for” scores as the expected prognosis decreased was insufficient (*p* for trend = 0.074). Compared to expected prognoses of ≥ 12 months, evidence that those of < six months were associated with lower “health and illness” scores (-13.5 points [95%CI -30.5 to 3.5]) was insufficient. Additionally, evidence for a decreasing trend in the “health and illness” scores as the expected prognosis decreased (*p* for trend = 0.229) was insufficient. Compared to expected prognoses of ≥ 12 months, evidence that those of six to 12 months and < six months were associated with lower “role and connectedness” scores was insufficient (2.8 points [95%CI -6.9 to 12.5] and -3.3 points [95%CI -16.4 to 9.8], respectively). Furthermore, evidence for a decreasing trend in the “role and connectedness” scores as the expected prognosis decreased was insufficient (*p* for trend = 0.901). Fig 2 and S6 Table present the association between expected prognosis and WHODAS 2.0 scores. Compared to expected prognoses of ≥ 12 months, those of six to 12 months and < six months were associated with higher WHODAS 2.0 scores (13.9 [95%CI 2.5–25.3] and 19.6 points [95%CI 4.3–34.8], respectively). Additionally, an increasing trend was observed in the WHODAS 2.0 scores as the expected prognosis decreased (*p* for trend = 0.002).

## Discussion

Among the patients receiving home medical care, short life expectancy was associated with lower hope for “something to live for,” although hope for “role and connectedness” was maintained. Additionally, short life expectancy was associated with higher QOL in relation to current home medical care, even though it was associated with decreased functioning.

The present findings on the relationship between life expectancy and QOL and hope in home medical care are consistent with previous findings in the palliative care field. First, the lower “something to live for” domain scores among patients with reduced life expectancy were consistent with previous studies reporting decreased hope for the future following perceived physical deterioration [9, 14]. Although not statistically replicable, lower hope scores for health were associated with shorter life expectancy in our study sample. This study combines our findings of lower perceived living function in patients with shorter life expectancy with the findings on hope regarding “health” and “something to live for.” This result suggests that home medical care patients with reduced life expectancy may suffer from loss of autonomy which includes physical independence, a sense of control over the future, and continuity of fulfilment in life) and temporality (which includes anxiety about death, recovery from illness, and hope for achieving goals) [29]. Second, the finding about hope for “role and connectedness,” which sustained even within six months of life expectancy, may indicate a benefit of receiving home medical care. This idea is supported by the maintenance of “connectedness with others” by a high percentage (approximately 90%) of patients with family members in this study; family or the other caregivers’ relationships are maintained through three-way communication between patients, their families or other caregivers, and home care physicians, and frequent visits by a multidisciplinary home medical care team [4]. In other words, the loss of relationships—including the desire to be with family and not be a burden to family members or health-care providers [29]—is minimized among patients receiving home medical care even if they have short life expectancies. Third, the unexpected relationship between short life expectancy and high QOL measured by the QOL-HC may be explained by the increased frequency of patients’ reflections on their life, which is part of this outcome measure. A report on improved QOL due to increased life reviewing near the end of life partially explains our findings [30]. Alternative explanations include improved patient satisfaction through interactions with home medical care providers and improved QOL caused by reduced pain and other symptoms through appropriate palliation [5, 31]. A further alternative explanation is a response shift to QOL-HC due to changes in internal standards and values accompanying repeated reflections on their lives [32]. In applying the results of this study to clinical practice, physicians should keep in mind that the patients might have lost their sense of purpose in life and hope for health. For example, it is important for home care physicians to recognize that patients may be losing hope for their future and their physical functions although they may appreciate their providers for care provided in their homes. Therefore, especially in the treatment of patients with limited life expectancy, physicians should have room for assessing patients’ psychological status and considering interventions to target their psychological state. For example, preparing home care physicians and their team nurses to provide spiritual care may be helpful in minimizing such loss of hope.

This study has several strengths. First, this study is the first to measure health-related hope by domain and correlate it with life expectancy among patients receiving home medical care. Second, our findings are generalizable because the study was conducted in a multicenter setting and the analyses accounted for differences in the clustering of outcomes across these facilities. Third, this study was designed so that the questionnaires were answered in the absence of the attending home medical care physician, and the forms were mailed directly to the central facility.

### Limitations

This study has some limitations. First, this study was conducted among patients who could answer the questionnaire independently. Therefore, patients with severe dementia and reduced consciousness, or those who may die within a few days were not included. Second, perceptions regarding the HR-Hope scale may vary across countries. While cultural and religious backgrounds and perceptions of spirituality may differ between Asian and Western countries [15], racial differences in tolerance of religious hope and psychological distress have also been reported [33]. Third, although this study assessed the presence of family members, it was not possible to assess whether family members lived with the patient or were caregivers. Since it was reported that the presence of family caregivers or full-time caregivers influences end-of-life care at home [34], future research is needed to investigate the details concerning family members. Fourth, although our previous study found that the proportion of patients who discussed advance care planning with their physicians was 29% [22], we did not examine whether the physicians informed them about their life expectancy, and thus could not assess its impact on patient’s HR-Hope and QOL-HC scores. Fifth, the COVID-19 outbreak during our study period may have affected the hope among patients receiving home medical care. However, because there were not many newly confirmed cases during that period (daily cases: median 55, interquartile range 24–266, maximum 1588) [35], we believe that its impact on patients’ hope was not dramatic.

## Conclusion

In summary, short life expectancy was associated with higher QOL concerning home medical care and with lower functioning and hope for “something to live for” among Japanese patients receiving home medical care. This study highlights the urgent need for home physicians to deliver a good end-of-life experience by focusing on hope.

## Supporting information

S1 Checklist STROBE statement—checklist of items that should be included in reports of observational studies. (DOCX)

S1 Table Quality of life for patients receiving home-based medical care. (DOCX)

S2 Table Japanese version of the Health-Related Hope Scale. (DOCX)

S3 Table The Japanese version of the World Health Organization Disability Assessment Schedule 2.0, 12-item version, self-administered. (DOCX)

S4 Table Associations between prognostic expectation and the Quality-of-Life Scale for Elderly Patients Receiving Professional Home Care (n = 194). (DOCX)

S5 Table Associations between expected prognosis, covariates, and the Health-Related Hope Scale domains (n = 197). (DOCX)

S6 Table Associations between prognostic expectation and the WHO Disability Assessment Schedule 2.0 (n = 194). (DOCX)

S1 Text Full details of the other members of the Zaitaku Evaluative Initiatives and Outcome Study Group. (DOCX)
