# Supplementary material for: Relationship of life expectancy with quality of life and health-related hope among Japanese patients receiving home medical care: The Zaitaku Evaluative Initiatives and Outcome Study
Source: PLoS One. 2023 Dec 14;18(12):e0295672. doi: 10.1371/journal.pone.0295672 (PMC10721024; doi:10.1371/journal.pone.0295672)
Supplement: S1 Table — (DOCX) [file pone.0295672.s002.docx]

**S1 Table. QOL for patients receiving home-based medical care. (QOL-HC).**

| Question 1 | おだやかな気持ちで過ごしていますか。  (English: “Do you have peace of mind?”) |
| --- | --- |
| Question 2 | 現在まで充実した人生だった、と感じていますか。  (English: “Do you feel satisﬁed with your life when you reﬂect on it?”) |
| Question 3 | 話相手になる人がいますか。  (English: “Do you have someone that you spend time talking with?”) |
| Question 4 | 介護に関するサービスに満足していますか。  (English: “Are you satisﬁed with the home care service system?”) |
| Response options for Question | いいえ(0) /どちらともいえない(1) /はい(2)  (English: Never agree (0)/Neither agree nor disagree (1)/always agree (2)) |

The original English version is also provided for each item and response.

**References**

[Kamitani H, Umegaki H, Okamoto K, et al. Development and validation of a new quality of life scale for patients receiving home‐based medical care: the Observational Study of Nagoya Elderly with Home Medical Care. *Geriatr Gerontol Int*. 2017;17(3):440-448. doi:](http://paperpile.com/b/Z2Jh4l/DgdCi)[10.1111/ggi.12735](http://dx.doi.org/10.1111/ggi.12735)
