# Supplementary material for: Relationship of life expectancy with quality of life and health-related hope among Japanese patients receiving home medical care: The Zaitaku Evaluative Initiatives and Outcome Study
Source: PLoS One. 2023 Dec 14;18(12):e0295672. doi: 10.1371/journal.pone.0295672 (PMC10721024; doi:10.1371/journal.pone.0295672)
Supplement: S2 Table — (DOCX) [file pone.0295672.s003.docx]

**S2 Table. Japanese version of the health-related hope scale (HR-Hope).**

| 過去30日間に，どれくらい難しさがありましたか。  In the past 30 days, how much difficulty did you have in: | |
| --- | --- |
| Question 1 | これからも楽しみを持ち続けることができると思う。  (English: “I think I’ll still be able to continue doing enjoyable things in the future.”) |
| Question 2 | 自分が生きる意味を見つけられるだろう。  (English: “I will probably be able to discover some meaning to my life.”) |
| Question 3 | 自分のできる範囲で、生きがいを見つけられるだろう。(English: “I will probably be able to work, to the best of my ability, to find a sense of purpose in life.”) |
| Question 4 | 毎日を大切に過ごしていけるだろう。  (English: “I will probably be able to live each day to its fullest.”) |
| Question 5 | 日々の暮らしの中で、生きがいを感じていけると思う。 (English: “I feel that I can continue to experience a sense of fulfillment in my daily life.”) |
| Question 6 | 病気によって気分が落ち込むことがあっても、気持ちを切り替えられるだろう。  (English: “Even if I should feel down due to my illness, I could probably turn my feelings around.”) |
| Question 7 | 病状に応じて、現実的な健康上の目標を決められると思う。(English: “I feel I can adjust my health goals in a way that is consistent with my actual disease condition.”) |
| Question 8 | 私は、病気や症状の変化に応じて、目標を修正できるだろう。 (English: “I can probably alter my goals depending on changes in my illness or symptoms”) |
| Question 9 | 病気によって目標が達成できなくなっても、また新たな目標を見つけられるだろう。  (English: “Even if my health condition keeps me from achieving my present goals, I will probably be able to find a new goal.”) |
| Question 10 | 病状に併せて、自分なりに生活の工夫ができるだろう。(English: “I can probably develop a personal lifestyle strategy for dealing with my disease condition.”) |
| Question 11 | 病気を悪化させないための方法を探すことができるだろう。 (English: “I will probably be able to find a way to keep my illness from worsening.”) |
| Question 12 | 現在、社会の中で果たしている役割をこれからも続けられるだろう。 (English: “I will probably be able to continue performing my role in society”) |
| Question 13 | 私の病気の体験を知ることで、周囲の人も健康を気遣うようになるだろう。  (English: “My disease experience will probably encourage those around me to be mindful of their own health.”) |
| Question 14 | 友人とより良い関係を作る事ができると思う。(English: “I feel I can deepen my relationships with my friends.”) |
| Question 15 | 私の周囲の人は私の気分転換に付き合ってくれるだろう。(English: “Those around me will probably go along with any changes in my mood.”) |
| Question 16 | 私の周囲の人はこれからも今まで通り接してくれるだろう。(English: “Those around me will probably continue to treat me the same way they always have”) |
| Question 17^a^ | 現在、家族の中で果たしている役割をこれからも続けられるだろう。(English: “I’ll probably be able to continue my usual role in support of my family.”) |
| Question 18* | 今後も家族との良い関わりが続くと思う。(English: “I feel that I’ll continue to have a good relationship with my family.”) |
| Response options  for Questions | 全くそう思わない(0) /少しそう思う(1) /ある程度そう思う(2) /とてもそう思う(3)  (English: I don’t feel that way at all (0)/ I feel that way a little (1)/ I feel that way somewhat (2)/ I feel that way strongly (3) |

^a^Questions for people with families

When using this instrument, please refer to the following reference.

**Reference**

Fukuhara S, Kurita N, Wakita T, Green J, Shibagaki Y.

A scale for measuring health-related hope: its development and psychometric testing.

Annals of Clinical Epidemiology 2019;1(3):102–119
