# Supplementary material for: Relationship of life expectancy with quality of life and health-related hope among Japanese patients receiving home medical care: The Zaitaku Evaluative Initiatives and Outcome Study
Source: PLoS One. 2023 Dec 14;18(12):e0295672. doi: 10.1371/journal.pone.0295672 (PMC10721024; doi:10.1371/journal.pone.0295672)
Supplement: S3 Table — (DOCX) [file pone.0295672.s004.docx]

**S3 Table. The Japanese version of the World Health Organization Disability Assessment Schedule 2.0 (WHODAS 2.0) 12-item version, self-administered.**

| 過去30日間に，どれくらい難しさがありましたか。  In the past 30 days, how much difficulty did you have in: | |
| --- | --- |
| Question 1 | 長時間（30分くらい）立っている  (Original: “Standing for long periods such as 30 minutes?”) |
| Question 2 | 家庭で要求される作業を行う  (Original: “Taking care of your household responsibilities?”) |
| Question 3 | 新しい課題、例えば初めての場所へ行く方法を学ぶ  (Original: “Learning a new task, for example, learning how to get to a new place?”) |
| Question 4 | 誰もができるやり方で地域社会の活動に加わるのに、どれほど問題がありましたか（例えば、お祭りや、宗教的又は他の活動）  (Original: “How much of a problem did you have joining in community activities (for example, festivities, religious or other activities) in the same way as anyone else can?”) |
| Question 5 | 健康状態のために、どれくらい感情的な影響を受けましたか(Original: “How much have you been emotionally affected by your health problems?”) |
| Question 6 | 何かをするとき、10分間集中する  (Original: “Concentrating on doing something for ten minutes?”) |
| Question 7 | １kmほどの長距離を歩く  (Original: “Walking a long distance such as a kilometer [or equivalent]?”) |
| Question 8 | 全身を洗う (Original: “Washing your whole body?”) |
| Question 9 | 全身で服を着る (Original: “Getting dressed?”) |
| Question 10 | 見知らぬ人に応対する  (Original: “Dealing with people you do not know?”) |
| Question 11 | 友人関係を保つ (Original: “Maintaining a friendship?”) |
| Question 12 | 毎日の仕事をする/学校へ行く (Original: “Your day-to-day work?”) |
| Response options for Question | 全く問題ない(1) /少し問題あり(2) /いくらか問題あり(3) /ひどく問題あり(4) /全く何もできない(5)  (Original: None (1)/Mild (2)/Moderate (3)/Severe (4)/Extreme or cannot do (5)) |

**References**

WHO. WORLD HEALTH ORGANIZATION DISABILITY ASSESSMENT SCHEDULE 2.0 12-item version, self-administered. <https://www.who.int/publications/i/item/measuring-health-and-disability-manual-for-who-disability-assessment-schedule-(-whodas-2.0)>

Accessed 29 September 2022

Tazaki M, Yamaguchi T, Yatsunami M, Nakane Y. Measuring functional health among the elderly: development of the Japanese version of the World Health Organization Disability Assessment Schedule II. Int J Rehabil Res. Mar 2014;37(1):48-53. DOI. 10.1097/MRR.0000000000000032
