# Supplementary material for: Relationship of life expectancy with quality of life and health-related hope among Japanese patients receiving home medical care: The Zaitaku Evaluative Initiatives and Outcome Study
Source: PLoS One. 2023 Dec 14;18(12):e0295672. doi: 10.1371/journal.pone.0295672 (PMC10721024; doi:10.1371/journal.pone.0295672)
Supplement: S4 Table — (DOCX) [file pone.0295672.s005.docx]

# **S4 Table. Associations between prognostic expectation and QOL-HC (n = 194).**

| QOL-HC, points | mean difference^a^ (95%CI) | P-value |
| --- | --- | --- |
| Expected prognosis |  |  |
| ≥ 12 months | Reference |  |
| ≥ 6 - < 12 months | 0.3 (-0.04 - 0.7) | 0.078 |
| < 6 months | **0.7 (0.1 - 1.3)** | **0.019** |
|  |  |  |
| Age, per 10y | 0.1 (-0.004 - 0.3) | 0.057 |
| Women vs. Men | 0.1 (-0.3 - 0.6) | 0.551 |
| Educational attainment |  |  |
| Junior high school or lower | Reference |  |
| High school | -0.18 (-0.7 - 0.3) | 0.486 |
| College/University/Graduate school/Other | -0.3 (-0.8 - 0.1) | 0.146 |
| Presence of family | 0.2 (-0.3 - 0.7) | 0.418 |
| Comorbidities |  |  |
| Cerebrovascular disease | 0.02 (-0.6 - 0.7) | 0.960 |
| Heart disease | 0.1 (-0.4 - 0.5) | 0.798 |
| Malignancy | -0.47 (-1.01 - 0.1) | 0.088 |
| Respiratory disease | 0.3 (-0.2 - 0.8) | 0.230 |
| Articular disease | -0.2 (-0.9 - 0.5) | 0.553 |
| **Dementia** | **0.6 (0.1 - 1.01)** | **0.015** |
| Neuromuscular disease | -0.2 (-0.98 - 0.5) | 0.528 |
| Fracture/Fall | -0.1 (-0.6 - 0.4) | 0.753 |
| Weakness | -0.14 (-0.6 - 0.3) | 0.558 |
| Spinal cord injury | 0.1 (-0.8 - 1.04) | 0.777 |

Analysis of 194 patients among 29 facilities.

^a^Mixed-effects linear regression models adjusted for covariates listed above with robust standard errors.
