# Supplementary material for: Relationship of life expectancy with quality of life and health-related hope among Japanese patients receiving home medical care: The Zaitaku Evaluative Initiatives and Outcome Study
Source: PLoS One. 2023 Dec 14;18(12):e0295672. doi: 10.1371/journal.pone.0295672 (PMC10721024; doi:10.1371/journal.pone.0295672)
Supplement: S5 Table — (DOCX) [file pone.0295672.s006.docx]

**S5 Table. Associations between expected prognosis, covariates, and HR-Hope domains**^*^ **(n = 197).**

|  | **Something to live for** |  | **Health and Illness** |  | **Role and connectedness** |
| --- | --- | --- | --- | --- | --- |
|  | mean difference, point estimate (95%CI) |  | mean difference, point estimate (95%CI) |  | mean difference, point estimate (95%CI) |
| Expected prognosis |  |  |  |  |  |
| ≥ 12 months | Reference |  | Reference |  | Reference |
| ≥ 6 - < 12 months | -1.2 (-13.6 to 11.1), p = 0.846 |  | 0.8 (-11.8 to 13.5), p = 0.9 |  | 2.8 (-6.9 to 12.5), p = 0.571 |
| < 6 months |  |  | -13.5 (-30.5 to 3.5), p = 0.12 |  | -3.3 (-16.4 to 9.8), p = 0.62 |
|  |  |  |  |  |  |
| Age, per 10y | -1.2 (-4.2 to 1.8), p = 0.433 |  | -2.4 (-5.5 to 0.7), p = 0.135 |  | -1.4 (-3.8 to 0.999), p = 0.25 |
| Women vs. Men | -0.96 (-8.8 to 6.9), p = 0.809 |  | 0.5 (-7.7 to 8.6), p = 0.91 |  | 1.6 (-4.7 to 7.9), p = 0.622 |
| Educational attainment |  |  |  |  |  |
| Junior high school or lower | -3.4 (-12.5 to 5.8), p = 0.468 |  | -4.02 (-13.5 to 5.5), p = 0.406 |  | -1.3 (-8.6 to 5.9), p = 0.716 |
| High school | **-11.3 (-20.6 to -2), p = 0.018** |  | -9.6 (-19.3 to 0.1), p = 0.052 |  | -6.5 (-13.9 to 0.998), p = 0.09 |
| College/University/Graduate school/Other | Reference |  | Reference |  | Reference |
| Presence of family | -5.04 (-15.8 to 5.7), p = 0.359 |  | -5.4 (-16.6 to 5.8), p = 0.345 |  | **6.4 (-2.3 to 15), p = 0.148** |
| Comorbidities |  |  |  |  |  |
| Cerebrovascular disease | -4.6 (-14.7 to 5.6), p = 0.377 |  | -5.01 (-15.5 to 5.5), p = 0.35 |  | **-9.2 (-17.3 to -1.04), p = 0.027** |
| Heart disease | 5.8 (-2.5 to 14), p = 0.172 |  | 3.6 (-5 to 12.1), p = 0.415 |  | 3.6 (-3.03 to 10.2), p = 0.288 |
| Malignancy | 7.9 (-4.4 to 20.2), p = 0.21 |  | 4.1 (-8.6 to 16.7), p = 0.529 |  | 0.4 (-9.3 to 10.2), p = 0.93 |
| Respiratory disease | -8.3 (-18 to 1.3), p = 0.09 |  | -4.8 (-14.8 to 5.2), p = 0.348 |  | -4.2 (-11.9 to 3.5), p = 0.286 |
| Articular disease | -4.8 (-15.7 to 6), p = 0.381 |  | -8.5 (-19.7 to 2.6), p = 0.134 |  | -4.4 (-13 to 4.2), p = 0.319 |
| Dementia | 4.9 (-5.1 to 15), p = 0.336 |  | 1.1 (-9.2 to 11.4), p = 0.831 |  | 7.2 (-0.8 to 15.1), p = 0.077 |
| Neuromuscular disease | -3.7 (-16.3 to 8.9), p = 0.562 |  | -9.8 (-22.8 to 3.3), p = 0.142 |  | -1.8 (-11.9 to 8.3), p = 0.726 |
| Fracture/Fall | 5.8 (-6.4 to 18), p = 0.354 |  | 7.95 (-4.6 to 20.5), p = 0.215 |  | 3.6 (-6.1 to 13.3), p = 0.465 |
| Weakness | -1.6 (-12.6 to 9.3), p = 0.77 |  | -2.6 (-13.9 to 8.7), p = 0.653 |  | 2.4 (-6.3 to 11.1), p = 0.587 |
| Spinal cord injury | -12.2 (-32.8 to 8.3), p = 0.243 |  | -9.9 (-31.1 to 11.3), p = 0.36 |  | -2.1 (-18.5 to 14.3), p = 0.801 |

Analysis of 197 patients among 29 facilities.

^*^Mixed-effects linear regression models adjusted for covariates listed above.
