# Supplementary material for: Relationship of life expectancy with quality of life and health-related hope among Japanese patients receiving home medical care: The Zaitaku Evaluative Initiatives and Outcome Study
Source: PLoS One. 2023 Dec 14;18(12):e0295672. doi: 10.1371/journal.pone.0295672 (PMC10721024; doi:10.1371/journal.pone.0295672)
Supplement: S6 Table — (DOCX) [file pone.0295672.s007.docx]

# **S6 Table. Associations between prognostic expectation and WHODAS 2.0**^*^ **(n = 194).**

| WHODAS 2.0, points | mean difference (95%CI) | P-value |
| --- | --- | --- |
| Expected prognosis |  |  |
| ≥ 12 months | Reference |  |
| ≥ 6 - < 12 months | **13.9 (2.5 - 25.3)** | **0.017** |
| < 6 months | **19.6 (4.3 - 34.8)** | **0.012** |
|  |  |  |
| **Age, per 10y** | **3.3 (0.5 - 6.1)** | **0.021** |
| Women vs. Men | 2.2 (-5.03 - 9.4) | 0.553 |
| Educational attainment |  |  |
| Junior high school or lower | Reference |  |
| High school | -5.2 (-13.7 - 3.3) | 0.232 |
| College/University/Graduate school/Other | 0.1 (-8.5 - 8.8) | 0.973 |
| **Presence of family** | **11.3 (1.2 - 21.3)** | **0.028** |
| Comorbidities |  |  |
| **Cerebrovascular disease** | **16.02 (6.6 - 25.4)** | **0.001** |
| Heart disease | -6.1 (-13.9 - 1.6) | 0.122 |
| Malignancy | -11.04 (-22.8 - 0.7) | 0.066 |
| Respiratory disease | 7.3 (-1.6 - 16.3) | 0.109 |
| Articular disease | 9.3 (-0.8 - 19.4) | 0.071 |
| Dementia | 4.5 (-4.9 - 13.9) | 0.347 |
| **Neuromuscular disease** | **16.4 (4.8 - 28)** | **0.006** |
| Fracture/Fall | 5.7 (-5.5 - 16.9) | 0.321 |
| Weakness | 3.02 (-7.1 - 13.2) | 0.560 |
| **Spinal cord injury** | **22.7 (3.8 - 41.6)** | **0.018** |

Analysis of 194 patients among 29 facilities.

^*^Mixed-effects linear regression models adjusted for covariates listed above.
