# Supplementary material for: Relationship of life expectancy with quality of life and health-related hope among Japanese patients receiving home medical care: The Zaitaku Evaluative Initiatives and Outcome Study
Source: PLoS One. 2023 Dec 14;18(12):e0295672. doi: 10.1371/journal.pone.0295672 (PMC10721024; doi:10.1371/journal.pone.0295672)
Supplement: S1 Text — (DOCX) [file pone.0295672.s008.docx]

**S7 Text. Full details of the other members of the ZEVIOUS Group.**

Shinsuke Muto^1^; Tatsunobu Natsubori^1,2^; Michiko Hinata^1,3^; Wataru Nakagawa^1^; Akihiko Yonenaga^1^; Lina Inagaki^1^; Shioto Itakura^1^; Nobuhiro Ikeda^1^; Tomoka Nakamura^1^; Naoya Miyashita^1^; Takuya Furugen^1^; Takafumi Abo^4,5^; Sadayuki Okudaira^4,6^; Kazuhiko Takuma^4,7^; Chihiro Tsuchiya^,8^; Masahiro Deguchi^4,9^; Takashi Fujii^4,10^; Yoshitaka Harada^4,11^; Seiji Matsuo^4,12^; Motomichi Nakagawa^4,13^; Ken Tanigawa^4,14^; Yoshio Ochi^4,15^; Sadanobu Ogasawara^4,16^; Kazuhiko Hoshino^4,17^; Momoko Aruga^18^; Yoshinori Nakamura^18^; Nobuhiro Sawa^19^; Yosuke Akashi^19^; Nobuyuki Miyagi^20^; Toyohiro Terasaki^21^; Kunihiro Kinoshita^22^; Masaji Kikukawa^23^; Hisakazu Kato^24^; Masayuki Amano^25^; Kentaro Asakura^26^; and Naoto Fukui^27^.

^1^You Home Clinic, Bunkyo City, Japan

^2^You Home Clinic Azumabashi, Sumida City, Japan

^3^You Home Clinic Azabudai, Minato City, Japan

^4^Dr. Net Nagasaki, Nagasaki City, Japan

^5^Abo Gastrointestinal Surgical Clinic, Nagasaki City, Japan

^6^Okudaira Geka, Nagasaki City, Japan

^7^Takuma Clinic, Nagasaki City, Japan

^8^Chihiro Naika Clinic, Nagasaki City, Japan

^9^Deguchi Surgery Clinic, Nagasaki City, Japan

^10^Fujii Surgical Clinic, Nagasaki City, Japan

^11^Harada Internal Medicine Clinic, Nagasaki City, Japan

^12^Nagasaki Takara Home Medical Care Clinic, Nagasaki City, Japan

^13^Nakagawa Surgical Clinic, Nagasaki City, Japan

^14^Tanigawa Clinic, Nagasaki City, Japan

^15^Ochi Clinic, Nagasaki City, Japan

^16^Nagasaki Memorial Hospital, Nagasaki City, Japan

^17^Hoshino Internal and Respiratory Medical Clinic, Nagasaki City, Japan

^18^Medical home care center, Tenri Hospital Shirakawa Branch, Tenri City, Japan

^19^Minami Nara General Medical Center, Oyodo, Japan

^20^Miyagi Clinic, Tenri City, Japan

^21^Terasaki Clinic, Nara City, Japan

^22^Kinoshita Clinic, Sakurai City, Japan

^23^Kikukawa Internal Medicine Clinic, Sakurai City, Japan

^24^Kato Clinic, Uda City, Japan

^25^Nosegawa Village National Health Insurance Clinic, Nosegawa, Japan

^26^Daifuku Clinic, Sakurai City, Japan

^27^Fukui Clinic, Uda City, Japan
